# Multidisciplinary Obstetric Simulation Training: Experience at KK Women’s and Children’s Hospital (KKH), Singapore, a Tertiary Referral Centre

**DOI:** 10.7759/cureus.55840

**Published:** 2024-03-09

**Authors:** Mingyue Li, Ann Wright, Lay Kok Tan, Manisha Mathur, Kok Hian Tan, Shephali Tagore

**Affiliations:** 1 Obstetrics and Gynecology, KK Women's and Children's Hospital, Singapore, SGP; 2 Maternal Fetal Medicine, KK Women’s and Children’s Hospital, Singapore, SGP; 3 Maternal Fetal Medicine, OBGYN DUKE-NUS Academic Clinical Program, KK Women’s and Children’s Hospital, Singapore, SGP; 4 Obstetrics and Gynecology, KK Women’s and Children’s Hospital, Singapore, SGP; 5 Maternal Fetal Medicine, KK Women's and Children's Hospital, Singapore, SGP

**Keywords:** obstetric anesthesia, emergency obstetric care, skills and simulation training, simulation in medical education, gynaecology and obstetrics

## Abstract

Background

Multidisciplinary simulation training in the management of acute obstetric emergencies has the potential to reduce both maternal and perinatal morbidity. It is a valuable tool that can be adapted for targeted audiences of different specialities at all experience levels from medical students to senior consultants.

Methods

In this study, pre- and post-course questionnaires of learners with varying levels of clinical experience from Obstetrics and Gynaecology (O&G), Anaesthesia, Neonatology, Emergency Medicine, midwifery, and nursing who undertook two simulation courses (namely the Combined Obstetrics Resuscitation Training course, CORE, and the CORE Lite), which comprised lectures and simulation drills with manikins and standardized patients, between 2015 and 2023 were compared. This also included a period when training was affected by the coronavirus disease 2019 (COVID-19) pandemic.

Results

The results showed that both simulation courses increased confidence levels among all learners in the management of obstetric emergencies.

Pre-course, participants were most confident in the management of neonatal resuscitation and severe pre-eclampsia, followed by postpartum haemorrhage. They were least confident in the management of vaginal breech delivery, uterine inversion, and twin delivery.

Post-course, participants were most confident in the management of neonatal resuscitation and shoulder dystocia, followed by postpartum haemorrhage. They were least confident in the management of uterine inversion and maternal sepsis, followed by vaginal breech delivery and twin delivery.

Whilst we saw a huge improvement in confidence levels for all obstetric emergencies, the greatest improvement in confidence levels was noted in vaginal breech delivery*, *twin delivery*, *and uterine inversion.

Conclusion

The simulation courses were effective in improving the confidence in the management of obstetric emergencies. While it may be difficult to measure the improvement in clinical outcomes as a result of simulation courses alone, the increase in confidence levels of clinicians can be used as a surrogate in measuring their preparedness in facing these emergency scenarios.

## Introduction

Simulation training has long been recognised as an invaluable tool in medical education. As early as 1898, a phantom pelvic model was introduced to simulate birth to provide residents with hands-on training opportunities at an annual meeting of the Association of American Medical Colleges (AAMC), when most deliveries were home-based [1]. More recently, studies have shown the benefits of multidisciplinary cross-speciality simulation training involving integrated acute obstetric interventions in improving the quality and safety of patient care [2-4] through heightened knowledge, better understanding, and team communication. While there is no replacement for real-life clinical experience, regular simulation training in obstetrics goes a long way to ensure skills are kept up to date. More importantly, the less experienced learners are exposed to complex obstetric scenarios and learn how to handle similar situations in real life. It is recommended that simulation training should be undertaken by anyone who may have a role in the acute management of any obstetric emergency including obstetric specialists and trainees, nurses, midwives, neonatologists, neonatal nurses, anaesthetists, emergency department (ED) physicians and even family medicine doctors [5].

It has also been established that team-based, rather than individual simulation training for rare but serious obstetric emergencies is the preferred approach [6] and a number of courses are available. The PRactical Obstetric Multi-Professional Training (PROMPT) course is a team-based training program which was established in 2008 with the aim of improving the ability of healthcare professionals of various disciplines to train for, manage and respond to acute obstetric emergencies. This program has been shown to reduce adverse patient outcomes and improve clinical outcomes [7]. After receiving formal training from the PROMPT Maternity Foundation trainers, local faculty members are accredited to conduct these courses. These courses can be adapted locally for training clinicians in their own maternity units, providing valuable multidisciplinary hands-on experience to all participants. 

Singapore Health Services (SingHealth) is the largest group of healthcare institutions in Singapore, consisting of tertiary hospitals and speciality centres as well as polyclinics which provide primary care island-wide. As part of the SingHealth cluster, KK Women’s and Children’s Hospital (KKH) and Singapore General Hospital (SGH) provide obstetric care to over 12,000 deliveries annually. As the largest maternity hospital, KKH also provides multidisciplinary care for high-risk obstetric cases and is the point of referral for complex cases. Appropriate training of healthcare staff is therefore particularly important to ensure that the ability to manage obstetric emergencies in this high volume busy obstetric unit is maintained.

A team of clinicians from KKH and SGH underwent training and obtained the PROMPT license in 2009 and since then multidisciplinary team simulation training has been conducted on a regular basis. After the course was modified to meet local needs, it was renamed the Combined Obstetric Resuscitation and Emergencies Training Project (CORE). This takes the form of didactic lectures and mock simulation training sessions involving high-fidelity models and standardized patients and is conducted three times a year by a dedicated team led by the senior obstetric faculty. The course targets obstetric specialist doctors and trainees, anaesthetists, midwives and labour ward nurses who are involved in the care of obstetric patients.

In December 2019, a global pandemic of coronavirus disease 2019 (COVID-19) resulted in limitations in face-to-face simulation training and the course had to be reformatted to ensure continuity in training while complying with the Ministry of Health’s guidelines for minimizing the risk of a disease outbreak amongst the multidisciplinary team trainers and participants. This led to the development of an adapted version of the CORE named CORE-Lite in 2020 on which lectures were conducted via an online platform and simulation training limited to five fully vaccinated persons per room (including the faculty) wearing masks.

The objective of this audit was to compare the confidence levels of participants in the management of obstetric emergencies based on their responses in the pre- and post-course questionnaires. 

## Materials and methods

Study design

This was a retrospective study comparing participants’ responses to an anonymised survey pre- and post-participation in the obstetric CORE and CORE Lite courses held between 2015 and 2023. Participants were surveyed regarding their confidence levels in handling various obstetric emergencies including severe pre-eclampsia, postpartum haemorrhage, breech delivery, maternal resuscitation, shoulder dystocia, uterine inversion and twin delivery on a scale of 1 to 5 (1 being strongly disagree, 5 being strongly agree). A sample of the survey is shown in the Appendix table. Results were collected and analysed using Microsoft Excel. 

Inclusion and exclusion criteria

All participants who participated in the obstetric CORE and CORE Lite courses held at our centre between 2015 and 2023 (an eight-year period) were included in this study. Incomplete responses and participants who registered but did not show up or complete the course were excluded from the study. 

Data collection

Two weeks prior to the simulation course, participants were given a survey to answer. The same survey was given to the participants two weeks post-course. Participants were asked a series of questions regarding their confidence levels in managing each obstetric emergency based on a 5-point Likert scale. Surveys are anonymised and data were collected and collated on Microsoft Excel. To ensure compliance with the pre- and post-course surveys, participants only received their certificate of participation after completing the course and the surveys. 

Statistical analysis

Based on the 5-point Likert scale, a response of confidence levels 4 and 5 was taken to be a positive result, 3 was neutral and 1-2 was taken to be negative. Results from the survey were analysed using a paired t-test via IBM SPSS Statistics for Windows, Version 23 (Released 2015; IBM Corp., Armonk, New York, United States). A p-value of <0.05 was taken to be significant. 

## Results

Results from all the CORE and CORE-Lite obstetric simulation courses held between December 2015 and April 2023 were analysed. The same survey was distributed to the participants before and after they attended their course. For each question, the participants were asked to answer on a scale of 1 to 5 from strongly disagree to strongly agree. Surveys were anonymized and results were analyzed via Microsoft Excel.

**Figure 1:**
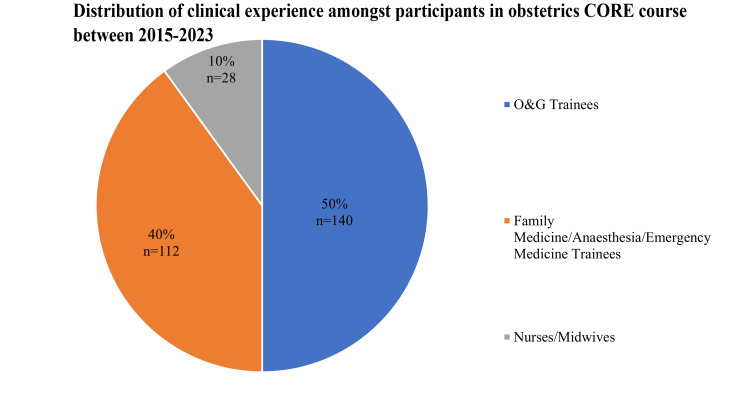
Distribution of clinical experience among participants in obstetrics (CORE) courses between 2015 and 2023 (n=280) CORE:  Combined Obstetric Resuscitation and Emergencies Training Project

A total of 280 participants took part in the courses and responded to the surveys during this period. The distribution of clinical experience among the participants is shown in Figure 1.

Participants came from varying backgrounds including local O&G trainees, nurses and midwives working at our maternal unity, as well as trainees from other disciplines and hospitals. Our Family Medicine, Anaesthesia and Emergency Medicine trainees may not be familiar with obstetric emergencies as they do not encounter these groups of patients on a regular basis. However, during the COVID-19 pandemic, we noted an increasing number of obstetric patients who presented to their Family Physicians or the EDs instead of our maternity unit. As a result, there was an increasing demand for simulation courses and training for these non-obstetric trainees and we started re-opening our courses to include these participants. 

**Figure 2:**
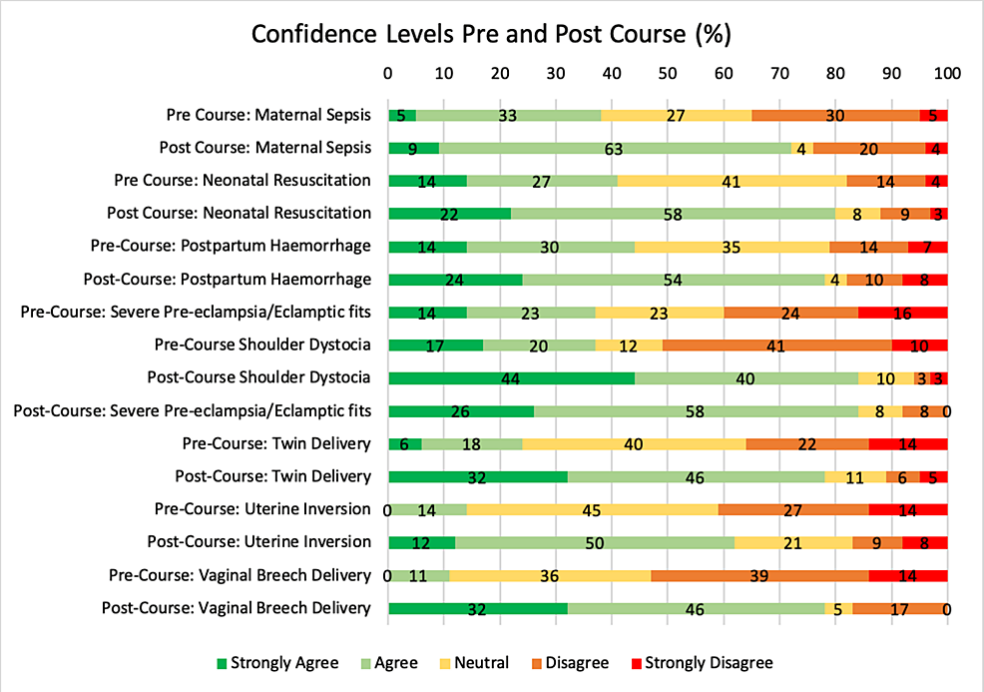
Confidence levels pre and post-course (%) Data is expressed in %

Participants were surveyed on their confidence levels in managing various obstetric emergencies including severe pre-eclampsia/eclamptic seizures, postpartum haemorrhage, breech delivery, neonatal resuscitation, maternal resuscitation, shoulder dystocia, and delivery of twins. The response rate for both pre- and post-course questionnaires was 100% and the post-course participant certificate was given only after completion of the surveys. A score of 4 or 5 was taken to be a positive confidence level, 3 was taken as neutral, whilst a score of 1-2 was taken to be a non-confident result based on a 5-point Liberty scale. The overall responses pre- and post-course are shown in Figure 2.

**Figure 3:**
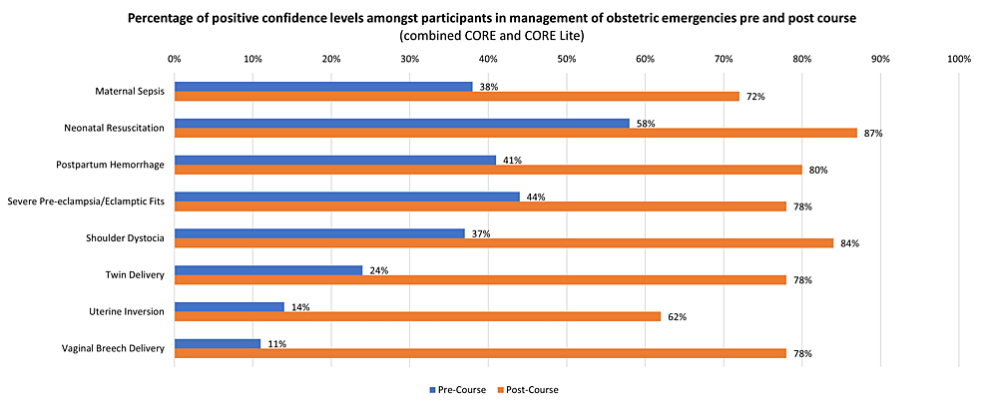
Percentage of positive confidence levels among participants in the management of obstetric emergencies pre- and post-course (combined CORE and CORE Lite) (n=280) CORE: Combined Obstetric Resuscitation and Emergencies Training Project

The overall increase in confidence levels in the management of various obstetric emergencies pre- and post-course is shown in Figure 3.

Results were analysed using the paired t-test and demonstrated a significant difference. This result indicated that there was an overall significant increase in confidence levels among participants in the management of obstetric emergencies post-simulation course. Results of the paired t-test analysis are shown in Table 1.

**Table 1:** Percentage of positive confidence level responses pre- and post-course

	Percentage of Positive Confidence Responses Pre-course (%)	Percentage of Positive Confidence Responses Post-course (%)	p-value	t-value
Maternal sepsis	38	72	0.00	8.57
Neonatal Resuscitation	58	87		
Postpartum Haemorrhage	41	80		
Severe Pre-eclampsia/Eclamptic fits	44	78		
Twin Delivery	37	84		
Uterine Inversion	24	62		
Vaginal Breech Delivery	11	78		

Our study showed an overall increase in confidence levels in the management of all obstetric emergencies after hands-on obstetric simulation courses. This increase in the confidence level was demonstrated in clinicians in different specialities, as well as obstetric nurses and midwives. 

From the results of the survey, we noted that prior to the course, participants were least confident in the management of vaginal breech delivery (n=31, 11%), followed by uterine inversion (n=39, 14%) and twin delivery (n=67, 24%). They were most confident in the management of neonatal resuscitation (n=162, 58%), severe pre-eclampsia (n=123, 44%), followed by postpartum haemorrhage (n=224, 80%). 

Post-course, participants were most confident in the management of neonatal resuscitation (n=243, 87%), shoulder dystocia (n=235, 84%​​​​​​​), followed by postpartum haemorrhage (n=224, 80%​​​​​​​). They were least confident in the management of uterine inversion (*n=172, *62%​​​​​​​*)*, maternal sepsis (n=201, 72%​​​​​​​), followed by vaginal breech delivery (n=218, 78%​​​​​​​), twin delivery (n=218, 78%), and severe pre-eclampsia (n=218, 78%).

While we saw a huge improvement in confidence levels for all the obstetric emergencies, the greatest improvement in confidence levels was noted in vaginal breech delivery (n=187, 67% increase​​​​​​​)*, *twin delivery (n=151, 54% increase​​​​​​​)*, *and uterine inversion (n=134, 48% increase​​​​​​​)*.*

## Discussion

Common examples of acute obstetric emergencies occurring in the Singhealth Obstetric Units, as in most labour wards, include pre-eclampsia, postpartum haemorrhage and unplanned vaginal breech delivery. Expeditious diagnosis and management of all these complications is important to ensure the best outcome for both mother and child.

Although obstetricians are not usually the first line in the management of neonatal resuscitation, it is a skill that is definitely useful in daily obstetric practice, particularly when neonatologists may not be available at delivery. As part of the O&G training program, residents are required to be regularly trained in neonatal resuscitation courses every two years and hold a valid neonatal resuscitation certificate. 

Pre-eclampsia is a common complication during pregnancy and is responsible for 10-15% of all maternal morbidity and mortality. The local incidence of severe pre-eclampsia is 29.3/10,000 deliveries [8]. Severe pre-eclampsia was one of the areas that participants were most confident in managing pre-course (44%, n=123). Timely recognition and management of severe pre-eclampsia can reduce the risk of maternal and fetal morbidity and mortality. Symptoms of pre-eclampsia may often overlap with other obstetric as well as non-obstetrics-related conditions (e.g. headaches, epigastric pains, blurring of vision). As such, when these patients present to clinicians who do not regularly manage obstetric emergencies, their diagnosis and treatment may be delayed, resulting in increased risks of maternal and fetal morbidity and mortality. 

Postpartum haemorrhage is the most common and dangerous complication of childbirth [9]. The incidence of postpartum haemorrhage in KKH is 5% [10], and 3.2% of women receive a blood transfusion either antenatally or in the postpartum period [11-13]. Causes of postpartum haemorrhage may include tone, trauma, tissue and thrombin. Uterine inversion is a potential cause for postpartum haemorrhage and should be considered as a diagnosis in the management of patients presenting with acute blood loss postnatally. Blood transfusion carries with it multiple risks and we aim to reduce to incidence of blood transfusion antenatally and in the postpartum period by picking up patients at risk of postpartum haemorrhage antenatally, by supplementing them with oral or parenteral iron antenatally to prepare them for labour. Patients who are at high risk of postpartum haemorrhage including grandmultiparity, borderline haemoglobin levels at the time of delivery, shoulder dystocia and multiple pregnancies are administered uterotonics at delivery. Postpartum blood loss may also be severely underestimated and timely recognition and escalation of care is essential in optimising outcomes. 

A breech presentation occurs in 3-4% of term deliveries [14]. Results of the term breech trial published in 2001 [15] discouraged many centres around the world including Singapore from routinely offering vaginal breech delivery which has led to a loss of skill in this practice. However, for mothers admitted in advanced labour with a breech presentation the option of vaginal birth delivery, conducted by a trained clinician, should be available. Patients with multiple pregnancies attempting vaginal delivery should be offered this option by a trained clinician so as to reduce the rates of caesarean sections, which carries risks in future pregnancies. 

We acknowledge that the main limitation of our study is that the improvement in confidence levels amongst clinicians in the management of obstetric emergencies may not be equivalent to an improvement in clinical outcomes. However, measurements of clinical outcomes are difficult to evaluate because of the vast number of variables and confounders involved. Due to the relatively low incidence of obstetric emergencies in daily practice, the collection of sufficient data for analysis will take a very long time. During this, new technologies, medications and innovative treatment may become available. Physicians who are at the front line taking care of obstetrics patients may also change their practice accordingly with advancing treatment and clinical experience. These confounders are difficult to account for in the evaluation of clinical outcomes pre and post-simulation courses, making such a study highly challenging to conduct. 

Another limitation of our study is the potential confounding variables including participants' experience levels. We acknowledge that individual participants in our obstetric simulation course may have different levels of clinical experience and prior training/courses which may be difficult to account for. However, there is a short interval between the conduction of surveys pre and post-course, and the survey was conducted amongst the same group of participants who took part in the same course. Therefore, confounders such as clinical experience and other courses/training are unlikely to interfere with our survey results. 

Using the confidence levels among clinicians in the management of the obstetric emergencies in this study should be considered a reasonable surrogate for the effectiveness of simulation courses. Previous studies have shown that simulation-based training programmes are effective at lowering risks of adverse outcomes in obstetric emergencies with a 38% reduction (2.1-1.3%) seen in the incidence of postpartum haemorrhage. They have also been associated with a positive influence on brachial plexus injuries from shoulder dystocia and maternal trauma with forceps and reduced early neonatal and 28-day mortality [4]. 

In our centre, the incidence of obstetric emergencies is audited monthly and recorded on the maternity dashboard. This reflects the department's compliance with international standards of maternal and obstetric care. Sentinel events are highlighted and reviewed internally within the department under a no-blame environment. Learning points from these reviews are disseminated to all members of the department via monthly bulletins and teaching sessions.

## Conclusions

While simulation courses will never be a substitute for real-life experience, our study showed that training in a controlled environment leads to a significant increase in confidence levels in the management of common obstetric emergencies. Regular courses are important for the maintenance of skills not only for obstetricians but also for other clinicians who may encounter obstetric emergencies in their day-to-day practice including those working as Emergency and Family Medicine clinicians.

Further courses for other challenging situations including complicated instrumental and difficult operative deliveries including second-stage Caesarean section are being developed at our centre as we continue training our future clinicians. Our goal is to improve and optimise care for each and every mother and child, and we will continue striving to work towards this goal at an international level. 

## Appendices

**Table 2:** Sample of pre- and post-course surveys

Maternal Sepsis	I am familiar with the diagnostic criteria suggestive of sepsis in a pregnant mother. I am aware of the elements of the ‘sepsis bundle’ used in the evaluation and treatment of maternal sepsis. I am familiar with the indications for transfer to Intensive Care Unit (ICU) in maternal sepsis. I am familiar with the steps of a perimortem Caesarean section	1= Strongly Disagree 2= Disagree 3= Neutral 4= Agree 5= Strongly agree 1= Strongly Disagree 2= Disagree 3= Neutral 4= Agree 5= Strongly agree
Neonatal Resuscitation	I am confident in my ability to lead the initial resuscitation of a newborn that is delivered flat at birth I am familiar with the position and technique for cardiac compressions and ventilation in neonatal resuscitation I am comfortable to assist my neonatal colleagues in the resuscitation of a newborn baby.	
Postpartum Hemorrhage	I am familiar with the ABCDE approach in dealing with severe post-partum hemorrhage I am familiar with the use of the 4Ts approach in the evaluation of severe postpartum hemorrhage. I am familiar with usage of medications in postpartum hemorrhage including the appropriate dosages to be used as well as their contraindications. I am confident in my clinical ability to estimate blood loss accurately.	
Severe Pre-eclampsia/Eclamptic Seizure	I am familiar with an ABCDE approach in dealing with a patient with severe pre-eclampsia I am familiar with the investigations that should be ordered in patients with severe pre-eclampsia I am confident in the use of medications (including the use of intravenous anti-hypertensives) in controlling high blood pressure in pregnant women. I am confident in the use of anti-convulsant (including the appropriate dilution) medications in eclamptic seizures. I appreciate the importance of teamwork in the management of eclamptic seizures.	
Shoulder Dystocia	I am confident in the steps used to achieve safe delivery of a baby in a case that is complicated by shoulder dystocia. I appreciate the importance of teamwork in the management of shoulder dystocia. I appreciate the importance of proper documentation in the management of cases complicated by shoulder dystocia.	
Twin Delivery	I am familiar with the necessary steps in the sequence of carrying out vaginal delivery of twins. I have an appreciation of the resources (manpower and equipment) needed to conduct safe vaginal delivery of twins. I am aware of the steps needed to determine the position of a non-cephalic second twin as well as the options for delivery such a twin.	
Uterine Inversion	I am familiar with the immediate management of acute uterine inversion. I am confident with the pharmacological treatment of the severe vasovagal response associated with uterine inversion. I am familiar with the pharmacological treatment of the severe vasovagal response associated with uterine inversion. I am familiar advanced techniques used in the replacement of an inverted uterus.	
Vaginal Breech Delivery	I am confident in the steps and manoeuvres required in delivery of a breech baby. I am familiar with the different methods used to deliver the aftercoming head in an assisted vaginal breech delivery. I am confident with the use of the Lovset’s manoeuvre to correct nuchal arm displacement in breech delivery.	
